# Tissue-plasminogen activator effects on the phenotype of splenic myeloid cells in acute inflammation

**DOI:** 10.1186/s12950-024-00375-0

**Published:** 2024-02-14

**Authors:** Célia Seillier, Léonie Lesec, Pauline Hélie, Charlotte Marie, Denis Vivien, Fabian Docagne, Brigitte Le Mauff, Olivier Toutirais

**Affiliations:** 1 Institut Blood and Brain @Caen-Normandie (BB@C), UMR-S U1237, Physiopathology and Imaging of Neurological Disorders (PhIND), Normandie Univ, UNICAEN, INSERM, GIP Cyceron, Caen, France; 2 UAR 3408-US50 / Centre Universitaire de Ressources Biologiques (CURB), GIP Cyceron, Caen, France; 3 Department of Clinical Research, Caen University Hospital, CHU Caen, France; 4 Department of Immunology and Histocompatibility (HLA), Caen University Hospital, CHU Caen, France; 5 Present address: Theodor Kocher Institute, University of Bern, Freiestrasse 1, CH-3012 Bern, Switzerland; 6 Present Address: INSERM, Département de L’information Scientifique Et de La Communication (DISC), 75654 Paris Cedex 13, France

## Abstract

Tissue-plasminogen activator (tPA) is a serine protease well known for its fibrinolytic function. Recent studies indicate that tPA could also modulate inflammation *via* plasmin generation and/or by receptor mediated signalling in vitro. However, the contribution of tPA in inflammatory processes in vivo has not been fully addressed. Therefore, using tPA-deficient mice, we have analysed the effect of lipopolysaccharide (LPS) challenge on the phenotype of myeloid cells including neutrophils, macrophages and dendritic cells (DCs) in spleen. We found that LPS treatment upregulated the frequency of major histocompatibility class two (MHCII^+^) macrophages but also, paradoxically, induced a deep downregulation of MHCII molecule level on macrophages and on conventional dendritic cells 2 (cDC2). Expression level of the CD11b integrin, known as a tPA receptor, was upregulated by LPS on MHCII^+^ macrophages and cDC2, suggesting that tPA effects could be amplified during inflammation. In tPA^−/−^ mice under inflammatory conditions, expression of costimulatory CD86 molecules on MHCII^+^ macrophages was decreased compared to WT mice, while in steady state the expression of MHCII molecules was higher on macrophages. Finally, we reported that tPA deficiency slightly modified the phenotype of DCs and T cells in acute inflammatory conditions. Overall, our findings indicate that in vivo, LPS injection had an unexpectedly bimodal effect on MHCII expression on macrophages and DCs that consequently might affect adaptive immunity. tPA could also participate in the regulation of the T cell response by modulating the levels of CD86 and MHCII molecules on macrophages.

**Supplementary Information:**

The online version contains supplementary material available at 10.1186/s12950-024-00375-0.

## Introduction

Inflammation is a host protective mechanism against invading microbial pathogens and tissue damage. It is a highly complex biological process and failure of regulatory mechanisms is associated with many diseases such as inflammatory bowel diseases, psoriasis or atherosclerosis [1–4].

Myeloid cells including neutrophils, macrophages and dendritic cells (DCs) are major actors of the inflammatory response. Through pattern recognition receptors (PRRs), they are able to sense microbial components and molecules derived from tissue damage [5]. Recognition of these danger signals leads to degranulation and reactive oxygen species production by neutrophils. In macrophages and DCs, activation of PRRs increase expression of major histocompatibility complex (MHC) and costimulatory molecules such as CD80 and CD86; and the production of pro-inflammatory cytokines that are required to activate adaptive immune response. Macrophages are a heterogeneous population of immune cells and schematically can polarise into two phenotypes: classical activated macrophages (M1) and alternatively activated macrophages (M2). M1 macrophages have a pro-inflammatory phenotype with pathogen-killing abilities and express markers such as inducible nitric oxide synthase (iNOS) and CD38, while anti-inflammatory M2 macrophages promote cell proliferation and tissue repair, and are defined by markers such as Arginase-1 and CD206 [6, 7].

DCs are considered as the most efficient antigen presenting cells capable of efficiently taking up and presenting antigens to naïve T cells, thus initiating the adaptive immune response. DCs also play a crucial role in maintenance of immune tolerance to self-antigens. While the lineage of DCs is diverse, conventional DCs (cDCs) expressing CD11c are the dominant subset in the spleen [8]. Using the integrin adhesion molecule CD11b, cDCs can be divided into two subtypes: cDC1 are MHCII^+^ CD11b^−^ whereas cDC2 have a MHCII^+^ CD11b^+^ phenotype. Specific functions of each subset are not completely understood but some reports suggest that cDC1 are highly specialised in cross-presentation and activation of cytotoxic T lymphocytes (CTL), whereas cDC2 preferentially stimulate helper T (Th) responses [9, 10].

In steady state, a pool of specialised DCs called “tolerogenic DCs” was found in the spleen of mice [11]. Tolerogenic DCs induce multiple mechanisms of immune tolerance, including T cell anergy or generation of peripheral regulatory T cells [12]. Tolerogenic DCs display an immature phenotype with low expression of MHCII and costimulatory molecules (CD80, CD86) on their surface [13].

Important bidirectional interactions exist between hemostasis and inflammation, two biological systems that are phylogenetically linked as host defence mechanisms [14–16]. Plasminogen (PLG)/plasmin system regulates the fibrinolysis and extracellular matrix degradation but also has diverse functions in inflammation [17–19]. This system comprises serine proteases, tissue-type plasminogen activator (tPA) and urokinase plasminogen activator (uPA) that cleave the PLG zymogen into plasmin, a key downstream enzyme that degrades fibrin. tPA activates PLG mostly within the vascular compartment while the proteolytic activity of uPA is regulated by its binding to the cell surface uPA receptor (uPAR) in tissues [17]. The PLG/plasmin system is tightly regulated at several levels. The PLG activators tPA and uPA are inhibited by the plasminogen activator inhibitors (PAI). The regulation of plasmin activity is tightly regulated by its major direct inhibitors, α2-antiplasmin and α2-macroglobulin (α2M) [17].

Beside their critical function in the fibrinolytic system, many reports indicate that tPA and uPA have also a complex role in inflammation and innate immunity [19–22]. Contrasting effects of PLG activators depend on their proteolytic activity or are mediated by a “cytokine-like” mode through interactions with specific receptors [23]. In vitro, tPA stimulates pro-inflammatory pathways *via* the generation of plasmin that induces production of pro-inflammatory cytokines by macrophages [24–26] but also through its interaction with annexin A2 receptor and CD11b co-receptor [27, 28]. However, tPA associated with α2M and Glucose-regulated protein-78 (Grp78) inhibits lipopolysaccharide (LPS)-induced-macrophage activation by interacting with the N-methyl-D-aspartate receptor (NMDA-R) and with the low density lipoprotein receptor-related protein-1 (LRP-1) [18, 29, 30].

Through the generation of plasmin, both uPA and tPA may also have a pro-inflammatory function. Interestingly, it is described that uPAR potentiates the LPS response *via* an interaction with the Toll-like receptor 4 (TLR4) [31]. PAI-1 is also involved in inflammatory responses and activates macrophages through TLR4 but independently of LPS [32].

In a murine *Escherichia coli* peritonitis model, tPA which is up-regulated in the liver and in the lung during infection has a protective effect [33]. tPA has also deleterious effects in inflammation. Indeed, Guo et al. showed that tPA-deficient mice had significantly higher rates of survival than WT mice in a model of sepsis induced by *Staphylococcus aureus* [34]. Paradoxically, Sugimoto et al. showed that pleural injection of PLG and plasmin induced a switch of macrophage polarisation toward a M2 phenotype suggesting that tPA or uPA may also contribute to the resolution of acute inflammation [35].

In the present study, the role of the tPA serine protease in acute inflammation was investigated by injecting LPS in tPA-deficient mice and analysing the distribution of phagocytes in the spleen. As expected, LPS increased the frequency of immunogenic macrophages (MHCII^+^ CD80^+^ CD86^+^ macrophages) but also, surprisingly, induced a downregulation of cell surface MHCII molecules on macrophages and cDC2. In addition, tPA deficiency limited LPS-induced CD86 expression on immunogenic MHCII^+^ macrophages and was associated with a higher MHCII expression on macrophages in steady state, suggesting a potential function of tPA in adaptive immunity. We also showed that tPA limited the expression of its own receptor, CD11b, on non-immunogenic MHCII^−^ macrophages.

## Materials and methods

### Mice

tPA^−/−^ (C57BL/6 J background) and control C57BL/6 J mice, aged 8–12 weeks, were provided by the *centre universitaire de ressources biologiques* (CURB, Normandy University, France). tPA^−/−^ mice were generated by an unique deletion of the exon-3 of the Plat gene, to avoid possible off target effect [36]. Mice were housed in our local conventional animal facilities at 21 °C in a 12 h light/dark cycle with food and water ad libitum. All procedures were performed according to the guidelines of the institutional ethics committee CENOMEXA (*comité normand d’éthique en matière d’expérimentation animale*). This protocol has been approved by this committee in accordance with the European directive n° 2013/63/UE (agreement number D14118001) and with the French and regional guidelines for animal experimentation and the use of genetically modified organisms (French Ministry of Research, project license #29,143).

### LPS Challenge

tPA^−/−^ mice were injected intraperitoneally with 1 mg/kg LPS (Sigma-Aldrich). Control mice received an equivalent volume of saline (NaCl 0.9%). 24 h later, mice were deeply anesthetised with 5% isoflurane (Aerrane, Baxter) and euthanised by cervical dislocation. The spleens and the blood were collected.

### Isolation of leukocytes from spleen

After mechanical disruption of the spleen, cell suspension was filtered through a 40 µm filter (Beckton Dickinson Biosciences) and erythrocytes were lysed with hypotonic buffer (0.8% NH_4_Cl, 0.1 mM EDTA, KHCO_3_, pH 7.4 [Stemcell Technologies]). Splenocytes were resuspended in Dulbecco’s modified Eagle medium (DMEM, Gibco) supplemented with 10% heat inactivated fetal bovine serum (FBS, Stemcell Technologies), 2.5% (v/v) HEPES (Fisher) and 1% penicillin/streptomycin (Gibco).

### Flow cytometry

Splenocytes were resuspended in 50μL of staining buffer and Fc receptors were blocked with 10 μg/mL anti-CD16/CD32 antibodies (Beckton Dickinson Biosciences) for 15 min at 4 °C. Cells were then labelled for cell surface markers with fluorochrome-conjugated monoclonal antibodies (Table 1) 10 min in the dark at 4 °C and 7-AAD (BioLegend) was added 15 min before analysis by flow cytometry. For intracellular staining, cells were fixed and permeabilised using “inside stain” kit according to manufacturer’s protocol (Miltenyi Biotec) before labelling with fluorochrome-conjugated monoclonal antibodies (Table 1). Samples were acquired on a FACSVerse (Beckton Dickinson Biosciences) and data analysed with the FlowJo 7.6.5 software (TreeStar Inc.).

**Table 1:** References of flow cytometry antibodies used for immunophenotyping

Target antigens	Clones	Fluorochromes	References	Isotypes/clones	Suppliers
CD16/32	2.4G2	-	553,142	-	BD
7-AAD	-	-	420,404	-	BioLegend
CD11b	REA592	VioBlue	130–113-810	human IgG1/REA293	Miltenyi Biotec
CD11c	REA754	APC	130–110-839	human IgG1/REA293	Miltenyi Biotec
F4/80	REA126	PE-Vio770	130–118-459	human IgG1/REA293	Miltenyi Biotec
CD80	REA983	APC-Vio770	130–116-463	human IgG1/REA293	Miltenyi Biotec
CD86	REA1190	PE	130–122-129	human IgG1/REA293	Miltenyi Biotec
MHCII	REA813	FITC	130–112-386	human IgG1/REA293	Miltenyi Biotec
CD38	REA616	PerCP-Vio700	130–109-260	human IgG1/REA293	Miltenyi Biotec
iNOS	REA982	APC	130–116-423	human IgG1/REA293	Miltenyi Biotec
CD71	REA627	PerCP-Vio700	130–109-577	human IgG1/REA293	Miltenyi Biotec
CD206	C068C2	APC	141,708	Rat IgG2a,k/RTK2758	BioLegend
Arg-1	A1EXF5	PE	12–3697-82	Rat IgG2a,k/eBR2a	Invitrogen

### Cytokine assay

Sera were collected 24 h following LPS injection and stored at -20 °C before cytokine assay using U-PLEX Biomarker Group 1 assays (K15069L-1) kit from Meso Scale Discovery (MSD). The cytokine assayed were IFN-γ, TNF, IL-10, IL-1β, IL-17, IL-4, IL-21 and MCP-1.

### Statistical analysis

Results are shown as the mean ± SD. Statistical analyses were performed using GraphPad Prism 9.0 software. To statistically compare four groups with two variables (treatment and genotype), we used an ordinary two-way ANOVA and when significant, a suitable Bonferroni multiple comparisons test was employed. To statistically compare two non-parametric groups, dependent on a single variable (genotype), *p*-values were calculated using the Mann–Whitney test. *P* < 0.05 is considered statistically significant. Only statistically significant *p-*values are reported in each graph. The ROUT method was used to determine outliers (Q = 1%).

## Results

### tPA deficiency did not modify neutrophil frequency in spleen after LPS challenge.

**Fig. 1:**
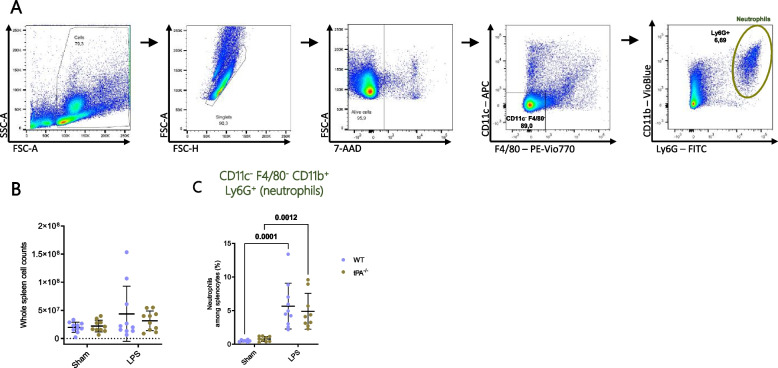
Increase of neutrophils in spleen after LPS challenge. **A** Representative flow cytometry gating strategy used for quantification of neutrophils among splenocytes (CD11c^−^ F4/80^−^ CD11b^+^ Ly6G^+^). **B** Cell number of total viable splenocytes by trypan blue exclusion on hemocytometer, *n* = 10/group. **C** Quantification of neutrophil frequency among splenocytes (Sham WT *n* = 7; Sham tPA^−/−^ *n* = 9; LPS WT *n* = 10; LPS tPA^−/−^ *n* = 9). Data are shown as individual animals with mean ± SD, two-way ANOVA with Bonferroni’s *post-hoc*

tPA has been described to induce human neutrophil migration and degranulation in vitro [37, 38]. So, we investigated by flow cytometry whether tPA deficiency could modify the frequency of neutrophils (CD11c^−^ F4/80^−^ CD11b^+^ Ly6G^+^) in inflammatory conditions after a 24 h LPS challenge. As previously described, neutrophils are poorly represented in the spleen [39]. The percentage of neutrophils is increased 24 h after LPS treatment (Fig. 1) but tPA deficiency did not modify the cellularity neither the frequency of neutrophils.

### ***Phenotype of WT and tPA***^***−/−***^*** splenic macrophages after LPS challenge***

**Fig. 2:**
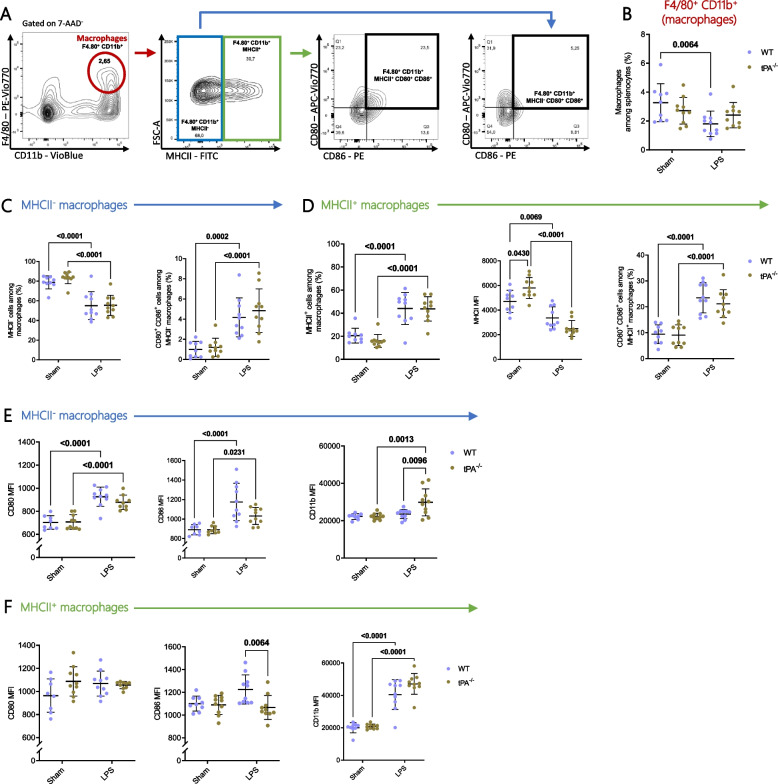
Effects of LPS treatment on the phenotype of splenic macrophages from tPA^−/−^ mice. **A** Representative flow cytometry gating strategy used for quantification of spleen macrophages (F4/80^+^ CD11b^+^), expressing MHCII molecules or not (F4/80^+^ CD11b^+^ MHCII^+or−^) and costimulatory molecules (F4/80^+^ CD11b^+^ MHCII^+or−^ CD80^+^ CD86^+^). **B** Frequency of macrophages (Sham WT *n* = 9; Sham tPA^−/−^; LPS WT; LPS tPA^−/−^ *n* = 10). **C** Frequency of MHCII^−^ macrophages (F4/80^+^ CD11b^+^ MHCII^−^) and of costimulatory molecule expressing cells (F4/80^+^ CD11b^+^ MHCII^−^ CD80^+^ CD86^+^), (Sham WT *n* = 9; Sham tPA^−/−^; LPS WT; LPS tPA^−/−^ *n* = 10). **D** Frequency of MHCII^+^ macrophages (F4/80^+^ CD11b^+^ MHCII^+^), (Sham WT *n* = 9; Sham tPA^−/−^; LPS WT; LPS tPA^−/−^ *n* = 10), MFI quantification of MHCII on MHCII^+^ macrophages (Sham WT *n* = 9; Sham tPA^−/−^; LPS WT; LPS tPA^−/−^ *n* = 10) and frequency of MHCII^+^ macrophages expressing costimulatory molecules (F4/80^+^ CD11b^+^ MHCII^+^ CD80^+^ CD86^+^), (Sham WT *n* = 9; Sham tPA^−/−^; LPS WT; LPS tPA^−/−^ *n* = 10). **E** MFI quantification of CD80 (Sham WT *n* = 8; Sham tPA^−/−^; LPS WT *n* = 10; LPS tPA^−/−^ *n* = 9), CD86 (Sham WT *n* = 8; Sham tPA^−/−^ *n* = 9, LPS WT; LPS tPA^−/−^ *n* = 10) and CD11b molecules (Sham WT *n* = 8; Sham tPA^−/−^; LPS WT; LPS tPA^−/−^ *n* = 10) on MHCII^−^ macrophages. **F** MFI quantification of CD80 (Sham WT *n* = 8; Sham tPA^−/−^; LPS WT *n* = 10; LPS tPA^−/−^ *n* = 9), CD86 and CD11b molecules (Sham WT *n* = 9; Sham tPA^−/−^; LPS WT; LPS tPA^−/−^ *n* = 10) on MHCII^+^ macrophages. Data are shown as individual animals with mean ± SD, two-way ANOVA with Bonferroni’s *post-hoc*

LPS challenge significantly decreased the frequency of macrophages (defined as F4/80^+^ CD11b^+^ cells) in spleen from WT mice but not tPA-deficient mice (Fig. 2A-B). By analysing macrophage activation markers, we observed that most of macrophages did not express MHCII molecules in sham condition whereas there was a significant increase of MHCII^+^ macrophage frequency after LPS treatment (Fig. 2C-D). Nevertheless, the level of MHCII molecules was decreased in macrophages after LPS treatment, in both WT and tPA^−/−^ mice (Fig. 2F). In addition, macrophages from tPA-deficient mice displayed a higher number of MHCII molecules on their surface, only in basal condition (median fluorescence intensity [MFI] Sham WT 4713 ± 900 *vs* Sham tPA^−/−^ 5797 ± 849, *p* = 0.0430).

The acute inflammation enhanced the expression of the CD80 and CD86 costimulatory molecules on macrophages. Indeed, the percentage of CD80^+^ CD86^+^ cells has more than doubled in both MHCII^−^ and MHCII^+^ splenic macrophages after exposure to LPS in WT and tPA-deficient mice (Fig. 2C-D). Regarding the cellular level of costimulatory molecules after LPS challenge, CD80 was upregulated in MHCII^−^ macrophages in WT and tPA^−/−^ mice (MFI Sham WT 703 ± 59 *vs* LPS WT 926 ± 83, *p* < 0.0001 and MFI Sham tPA^−/−^ 708 ± 63 *vs* LPS tPA^−/−^ 878 ± 63, *p* < 0.0001) but remained stable on MHCII^+^ macrophages (Fig. 2E-F). In MHCII^−^ but not in MHCII^+^ macrophages, CD86 expression levels were also raised after LPS treatment in both mouse strains (Fig. 2E-F). In MHCII^+^ macrophages, we observed that CD86 expression was higher in WT compared to tPA-deficient animals under LPS condition (MFI LPS WT 1226 ± 129 *vs* LPS tPA^−/−^ 1067 ± 105, *p* = 0.0064; Fig. 2F).

After LPS injection, there was an increase of CD11b integrin expression on MHCII^+^ macrophages regardless of the mouse strains (Fig. 2F). By contrast, in MHCII^−^ macrophages, CD11b is significantly upregulated only in tPA-deficient mice under inflammatory (MFI Sham tPA^−/−^ 22,264 ± 1738 *vs* LPS tPA^−/−^ 29,841 ± 7236, *p* = 0.013 and MFI LPS WT 23,549 ± 2511 *vs* LPS tPA^−/−^ 29,841 ± 7236, *p* = 0.0096; Fig. 2E). This result suggested an inhibitory role of tPA on the induction of CD11b by LPS.

Although CD11c is described as a DC marker, many authors have reported the expression of this integrin on macrophages in both lymphoid and non-lymphoid tissues [40, 41]. Unlike the total macrophages, CD11c^+^ macrophage frequency was significantly increased in spleen after LPS treatment, in both mouse genotypes (Supplementary data 1A and B). Notably, CD11c expression level was downregulated in acute inflammation context (Supplementary data 1B). As observed in total MHCII^+^ macrophages, LPS treatment increased CD11b level expression in CD11c^+^ MHCII^+^ macrophages with similar expression levels in each group (Fig. 2F and Supplementary data 1D). In CD11c^+^ MHCII^−^ macrophages, CD11b levels were increased in LPS condition in both genotypes, with an additional tPA effect (Supplementary data 1C). So, as seen in total MHCII^−^ macrophages, tPA limited LPS effect on CD11b expression.

As observed in total macrophages, there was a decrease of MHCII levels in CD11c^+^ macrophages upon LPS treatment in both WT and tPA-deficient mice (Fig. 2F and Supplementary data 1D). After LPS treatment, CD80 expression level was stronger on MHCII^+^ and MHCII^−^ CD11c^+^ macrophages in both WT and tPA-deficient mice. CD86 expression level was raised on MHCII^+^ CD11c^+^ macrophages from both genotypes of mice, but only on tPA-deficient mice for MHCII^−^ CD11c^+^ macrophages (Supplementary data 1C-D).

### M1/M2 macrophage polarisation in WT and tPA^−/−^ mice after LPS challenge

**Fig. 3:**
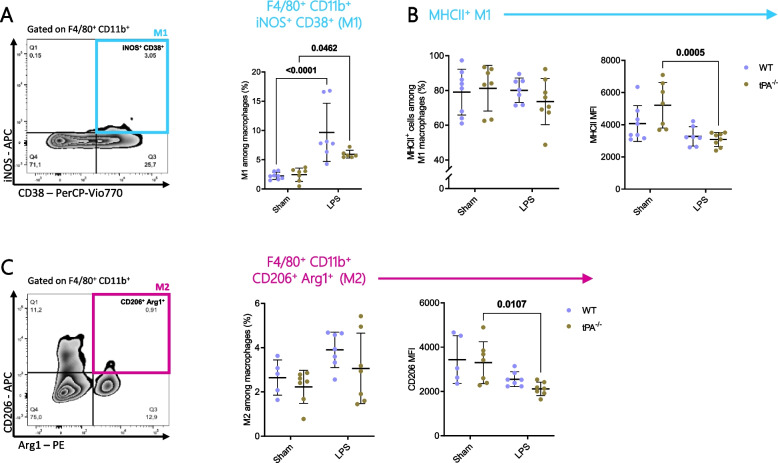
M1/M2 macrophage polarisation following LPS challenge. **A** Dot plot analysis of iNOS and CD38 expression for quantification of M1 frequency (F4/80^+^ CD11b^+^ iNOS^+^ CD38^+^), (Sham WT *n* = 8; Sham tPA^−/−^; LPS WT *n* = 7; LPS tPA^−/−^ *n* = 6). **B** Frequency of MHCII^+^ M1 macrophages and MFI of MHCII molecules on M1 (Sham WT *n* = 8; Sham tPA^−/−^; LPS WT *n* = 7; LPS tPA^−/−^ *n* = 8). **C** Dot plot analysis of CD206 and Arg-1 expression for quantification of M2 frequency (F4/80^+^ CD11b^+^ CD206^+^ Arg-1^+^) and MFI of CD206^+^ molecules (Sham WT *n* = 5; Sham tPA^−/−^; LPS WT; LPS tPA^−/−^ *n* = 7). Data are shown as individual animals with mean ± SD, two-way ANOVA with Bonferroni’s post-hoc

Since it was reported that tPA could influence M1/M2 macrophage polarisation in chronic renal disease [28, 42], we examined the impact of tPA deficiency in the differentiation state of splenic macrophages upon acute inflammation. LPS challenge induced an increase of M1 macrophages (iNOS^+^ CD38^+^) in WT and tPA-deficient mice (Fig. 3A). In addition, the MHCII level was significantly decreased in tPA-deficient mice after LPS stimulation as compared to control mice (Fig. 3B).

We did not notice any modification of M2 macrophage (CD206^+^ Arg1^+^) frequency in inflammatory condition with or without tPA (Fig. 3C). However, expression level of CD206 is significantly reduced in tPA^−/−^ but not in WT mice after LPS treatment.

### Splenic cDC distribution following LPS challenge

**Fig. 4:**
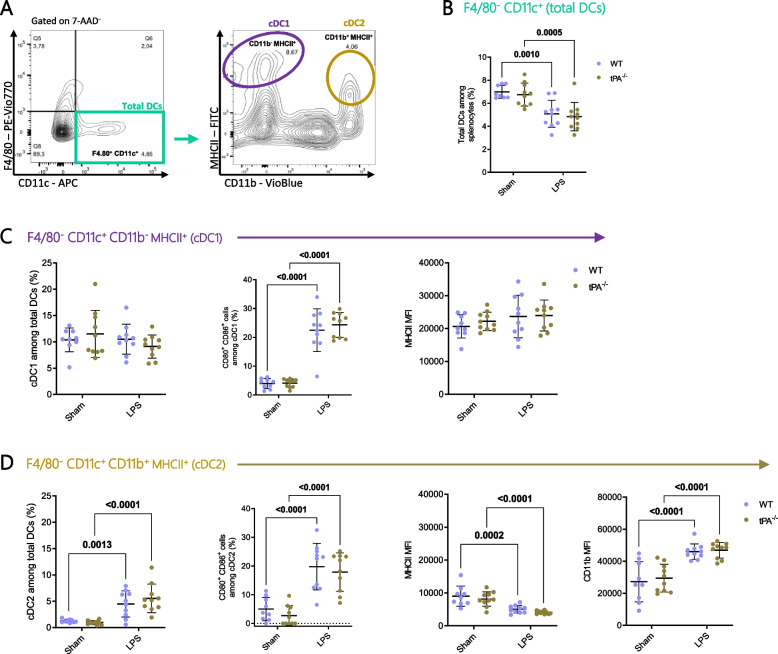
cDC phenotype was modulated in an inflammatory setting. **A** Representative flow cytometry gating strategy used for quantification of total DCs (F4/80^−^ CD11c^+^) and cDCs: cDC1 (F4/80^−^ CD11c^+^ CD11b^−^ MHCII^+^) and cDC2 (F4/80^−^ CD11c^+^ CD11b^+^ MHCII^+^). **B** Quantification of total DC frequency (Sham WT *n* = 8; Sham tPA^−/−^; LPS WT; LPS tPA^−/−^ *n* = 10). **C** Frequency of cDC1, MFI of MHCII molecules on cDC1 and frequency of CD80^+^ CD86^+^ cDC1 (Sham WT *n* = 9; Sham tPA^−/−^; LPS WT; LPS tPA^−/−^ *n* = 10). **D** Frequency of cDC2 and CD80^+^ CD86^+^ cDC2, MFI of MHCII and CD11b on cDC2 (Sham WT *n* = 9; Sham tPA^−/−^; LPS WT; LPS tPA^−/−^ *n* = 10). Data are shown as individual animals with mean ± SD, two-way ANOVA with Bonferroni’s post-hoc

A decrease of total DCs (F4/80^−^ CD11c^+^) was observed in spleen from WT and tPA^−/−^ mice treated with LPS (Fig. 4A and B). In LPS-challenged WT or tPA^−/−^ mice, the frequency of cDC1 (CD11b^−^ MHCII^+^) was not modified while the frequency of cDC2 (CD11b^+^ MHCII^+^) was increased in tPA^−/−^ and WT mice (Fig. 4C and D).

cDC1 expressed a higher level of MHCII molecules than cDC2 or macrophages, whatever the experimental conditions. The level of MHCII molecules on cDC1 was not modified by LPS, while on cDC2 LPS reduced MHCII expression in both strains (Fig. 4C-D).

In steady state, the costimulatory molecules CD80 and CD86 were weakly expressed in both cDC subsets. LPS treatment increased the frequency of CD80^+^ CD86^+^ cells in both cDC1 and cDC2 at the same level as MHCII^+^ macrophages (about 25% of CD80^+^ CD86^+^ cells after LPS injection; Fig. 4C-D).

We distinguished three MHCII^−^ tolerogenic DC subsets according to the CD11b level. The two subsets CD11b^−^ MHCII^−^ and CD11b^low^ MHCII^−^ DCs were not quantitatively modified by LPS treatment in neither WT nor tPA^−/−^ mice (Supplementary data 2A-C) whereas the third one (CD11b^+^ MHCII^−^ DCs) was raised upon inflammatory stimulation only in tPA^−/−^ mice (Supplementary data 2D). The CD11b^+^ subset was the less abundant but displayed the higher proportion of CD80^+^ CD86^+^ cells after LPS treatment. When comparing the costimulatory molecule expression magnitude, CD11b^−^ and CD11b^low^ tolerogenic DCs expressed lower levels of CD80 molecules as compared to the CD11b^+^ subset, even in inflammatory context (Supplementary data 3). The CD11b^−^ subset expressed the higher level of CD86 molecules after LPS stimulation. As observed in MHCII^+^ macrophages, the level of CD11b expression was increased in CD11b^low^ after LPS treatment but not in CD11b^+^ tolerogenic DCs (Supplementary data 2 and Fig. 2F).

### tPA modulated T cell activation after LPS treatment

**Fig. 5:**
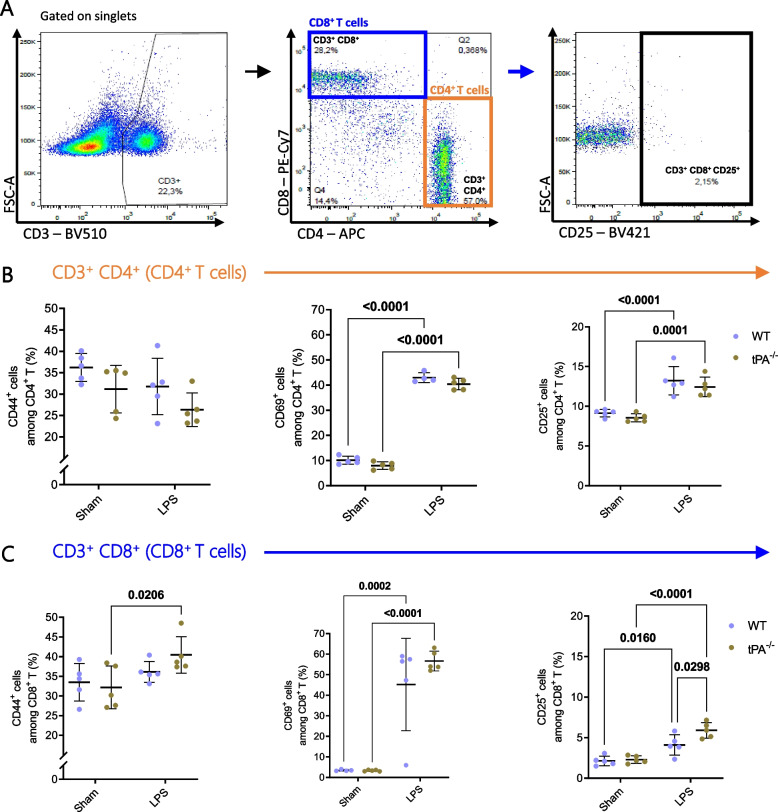
tPA modulated T cell activation during inflammation. **A** Dot plot analysis of CD3, CD4 and CD8 expression for quantification of T cell frequency (CD3^+^ CD4^+^ T or CD3^+^ CD8^+^ T) and CD44, CD69, CD25 for activated CD4^+^ or CD8^+^ T cells (CD3^+^ CD4^+^ CD44^+^/CD69^+^/CD25^+^ and CD3^+^ CD8^+^ CD44^+^/CD69^+^/CD25^+^). **B** Frequency of CD4^+^ T expressing CD44 (*n* = 5/group), CD69 (Sham WT; Sham tPA^−/−^ *n* = 5; LPS WT *n* = 4; LPS tPA^−/−^ *n* = 5) and CD25 (*n* = 5/group) activation markers. **C** Frequency of CD8^+^ T expressing CD44 (*n* = 5/group), CD69 (Sham WT *n* = 4; Sham tPA^−/−^; LPS WT; LPS tPA^−/−^ *n* = 5) and CD25 (*n* = 5/group) activation markers. Data are shown as individual animals with mean ± SD, two-way ANOVA with Bonferroni’s *post-hoc*

Few data exist on the effects of LPS injection on splenic T cell activation in vivo. LPS increased the frequency of CD69^+^ and CD25^+^ in both CD4^+^ and CD8^+^ T cells, and in both mice strains (Fig. 5A-C). In addition to LPS effect, tPA had an intrinsic and inhibitory effect on CD8^+^ CD25^+^ T cells (*p* = 0.0298). LPS didn’t have any effect on CD4^+^ CD44^+^ whereas it increased the frequency of CD8^+^ CD44^+^ population in tPA-deficient mice (Fig. 5C).

### tPA deficiency did not modify the cytokine secretion profile after LPS treatment

**Fig. 6:**
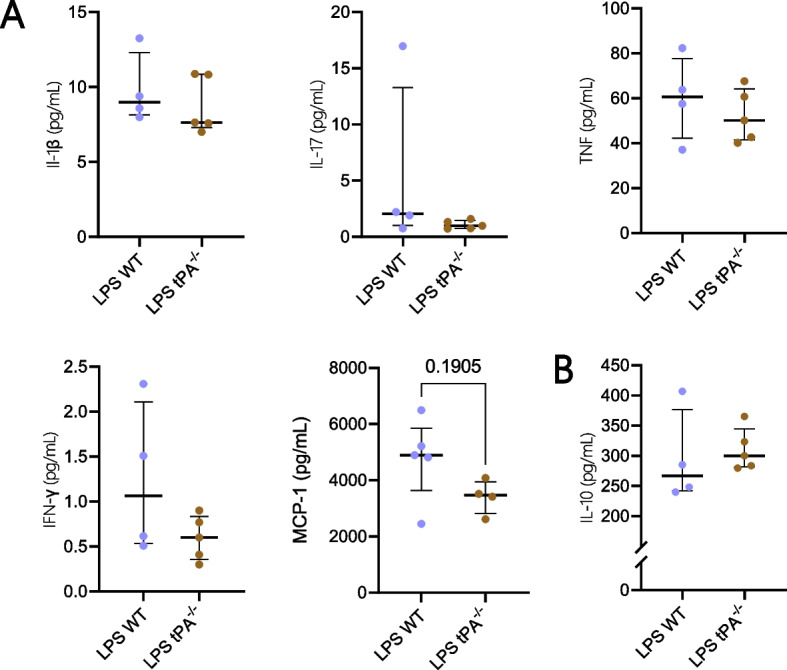
tPA did not affect serum cytokine production in LPS condition. **A** Pro-inflammatory cytokines: IL-1β, IL-17, TNF, IFN-γ (LPS WT *n* = 4; LPS tPA^−/−^ *n* = 5) and MCP-1 (LPS WT *n* = 5; LPS tPA^−/−^ *n* = 4) measurement in mouse sera. **B** Anti-inflammatory cytokine: IL-10 (LPS WT *n* = 4; LPS tPA^−/−^ *n* = 5) measurement in mouse sera. Data are shown as individual animals with mean ± SD, Mann–Whitney test

We have investigated the effects of tPA on the systemic cytokine secretion upon acute inflammation. Production of IL-1β, TNF, MCP-1 and IL-10 but not IFN-γ and IL-17 was detected in the sera of LPS-treated mice (Fig. 6). No significant differences were noted between WT and tPA^−/−^ mice.

## Discussion

Previous studies have shown that tPA is a modulator of inflammation that, for instance, regulates the biology of macrophages [29, 43]. However, little information exists about the role of the serine protease in inflammatory processes in vivo. Although LPS has been widely used to stimulate macrophages in vitro, its impact in vivo on splenic myeloid cell phenotype has been poorly studied. In resting state, a small population of splenic macrophages expressing MHCII and costimulatory molecules harbour an « immunogenic» phenotype. In an original way, we showed that LPS treatment upregulated the frequency of MHCII^+^ macrophages but also dramatically decreased the MHCII molecule spleen’s expression level in vivoin vivo [44]. Although it has been reported that in vitro stimulation with LPS increased MHCII^+^ frequency and expression [45], it has also been described that LPS antagonises the stimulating effect of IFN-γ or IL-4 on MHCII molecule expression in vitro [46–48]. The inhibitory pathway triggered by LPS is currently unknown but could involve autocrine or paracrine secretion of IL-10 by macrophages [49], a cytokine known to downregulate MHCII expression. On the other hand LPS-mediated inhibitory effect on MHCII expression, may explain why LPS impedes T cell-mediated response in experimental autoimmune encephalomyelitis (EAE) [50].

In a previous study, we have shown that, in vitro, tPA increased the level of MHCII molecules on splenic macrophages and DCs from EAE mice [51]. Here, in steady state, we observed a higher level of MHCII molecules on macrophages in tPA^−/−^ mice, probably reflecting a more complex and multifactorial regulation of these molecules in vivo.

Lin et al. have shown that CD11b is a co-receptor of annexin A2 involved in the tPA intracellular signalling [27]. Importantly, we evidenced that LPS strongly increased the level of CD11b on MHCII^+^ macrophages, cDC2 and tolerogenic CD11b^low^ DCs suggesting that the Gram^−^ endotoxin could increase the sensitivity of these cell subsets to tPA. Of note, upregulation of CD11b by LPS is observed in total MHCII^−^ and CD11c^+^ MHCII^−^ macrophages from tPA^−/−^ but not in WT mice. It could be a negative feedback of tPA, on one of its receptors, regulating tPA activity in these macrophages in inflammatory conditions. In addition, CD11b was also described as a fibrinogen/fibrin receptor able to promote macrophage activation [52].

Although the M1/M2 polarisation paradigm mainly arose from in vitro studies [53, 54], our results showed that LPS injection induced an upregulation of M1 cells in vivo. Frequency of M1 cells was not significantly different in tPA^−/−^ mice compared to WT mice after LPS treatment indicating that tPA is not involved in M1 polarisation in our acute model of inflammation. Previous studies have shown that tPA promotes M1 phenotype in vitro and in a model of chronic renal disease in vivo, suggesting that tPA might differently modulate the M1 phenotype in acute *versus* chronic inflammation [28]. The role of tPA in regulation of macrophage activation is nevertheless more complex since it also maintains the expression of the M2 marker CD206 after LPS stimulation without modifying the frequency of M2 cells. Sugimoto et al. have shown that pleural injection of PLG and plasmin increased CD206 expression on macrophages suggesting that tPA may have a pleiotropic and dynamic role in the course of inflammation acting both at the beginning and resolving phases of inflammation [35].

Splenic CD11c^+^ macrophages are associated with inflammatory pathologies in organs such as lung and kidney [40, 41]. In our study, this subset retains many phenotypic characteristics of MHCII^+^ macrophages but are more prone to express CD80 and CD86 costimulatory molecules. Drutman et al. have shown that splenic CD11c^+^ macrophages also share close phenotypic and functional properties with activated macrophages (endocytosis and poor T cell stimulation ability) [55]. Our results also confirmed previous observation showing that LPS treatment decreases CD11c expression on myeloid cells [56]. It has been suggested that CD11c/CD18, acting as a LPS receptor, is downregulated following the binding with its ligand [57].

In our study, LPS treatment reduced the proportion of total cDCs in spleen, likely by altering their migratory capacities and no effect of tPA on the frequency of total cDCs, neither cDC1 and cDC2 subsets was noticed. These results are not in agreement with Draxler et al. who have described that tPA induced a decrease of splenic cDC proportion 24 h after its administration in mice [58].

Hancock et al. have shown that cDC1 and cDC2 isolated from LPS-treated mice have distinct transcriptomic signatures even though they share a set of common genes induced by LPS [59]. Although the cDC subsets have redundant functions, cDC1 are specialised in CTL and Th1 responses while cDC2 are more prone to regulate T cell differentiation towards Th2 or Th17 profiles [60]. Even though no effect of tPA deficiency on cDC1 or cDC2 phenotype was observed in the present study, further investigations are needed to know if tPA may modify the cDC functions such as endocytosis or cytokine secretion, and so, may impact the outcome of the T cell response. Indeed, Borg et al. have shown that plasmin increases the phagocytic ability of mouse or human DCs in vitro [61].

We have distinguished three populations of tolerogenic MHCII^−^ DCs. Interestingly, we noticed that these DC subsets expressed various levels of CD80 and CD86 molecules. CD80 was weakly expressed at steady state and was modestly increased after LPS challenge, contrary to CD86. In addition, we highlighted that tPA upregulated CD86 expression on MHCII^+^ macrophages. Regarding our observation, Vago et al. have shown that after in vitro IFN-γ and LPS stimulation of M1-like bone-marrow-derived macrophages (BMDMs) deficient for the PLG receptor PLG-R_KT_ expressed a higher level of CD86 molecules while M1-like BMDMs treated with PLG and plasmin expressed a lower level of CD86 molecules. These findings suggest that the interaction of PLG with its receptor leads to the downregulation of CD86 in an inflammatory context [62]. A possible explanation for these observations is that tPA could decrease the PLG bioavailability and thus limit the decrease of CD86 on activated macrophages. The relative contribution of these two costimulatory molecules in the immune response was not completely understood. CD80 and CD86 molecules played a differential role in a mouse sepsis model after caecal ligation and puncture (CLP) [63]. Indeed, by using CD80- or CD86-deficient mice, authors have shown that CD80, but not CD86 is associated to an enhanced inflammatory response in vivo and decreased survival after CLP. Moreover, it was shown that the two costimulatory molecules have an opposite role in regulatory T cell immunosuppressive functions with an inhibitory role for CD86 and an activating role for CD80 [64]. So, it is possible that tPA may have an immunomodulatory activity in vivo.

We observed that almost half of splenic T cells are activated 24 h after LPS challenge. CD69 was the activating marker that is the most upregulated for both CD4^+^ and CD8^+^ T cells. tPA slightly decreased the activation of CD8^+^ T cells.

The moderate effects of tPA deficiency on inflammatory response observed in our study, including mainly modulation of CD86 and MHCII molecule expression on macrophages, may be explained by several hypothesis. First, as previously described, tPA has pro-and anti-inflammatory effects that may neutralise each other in vivo. In a non-exclusive manner, other molecules of the PLG/plasmin system involved in the regulation of inflammation may compensate the tPA deficiency. For example, some pro-inflammatory effects of tPA due to plasmin generation may be carry out by uPA. Comparison of single mice lacking tPA or uPA with doubly deficient mice lacking both tPA and uPA may be relevant to decipher the respective contribution of PLG activators in acute inflammation. With the use of tPA^−/−^;uPA^−/−^ mice, it has been demonstrated that plasmin was deleterious during *Staphylococcus aureus* infection in mice due to high levels of inflammatory cytokine production [34].

In conclusion, our study updates the knowledge on effects of LPS on the mononuclear phagocyte phenotype in vivo and mainly reports on its contrasted function in inflammation. On one hand, LPS increases the proportion of immunogenic macrophages expressing MHC class II and costimulatory molecules. On the other hand, it decreases the MHCII expression level and so, reduces potentially their ability to stimulate T cells in an efficient way. tPA has a slight effect in our model but further investigation is required to evaluate if tPA has a differential function in acute *versus* chronic inflammation and if it has a putative role in adaptive immune response in vivo.

### Supplementary Information

**Additional file 1.**

## Data Availability

Raw and analysed data, resources and reagents used in this study are available from the corresponding author upon request.
