# Supplementary material for: Tissue-plasminogen activator effects on the phenotype of splenic myeloid cells in acute inflammation
Source: J Inflamm (Lond). 2024 Feb 14;21:4. doi: 10.1186/s12950-024-00375-0 (PMC10865617; doi:10.1186/s12950-024-00375-0)
Supplement: Supplementary file 1 — Additional file 1. [file 12950_2024_375_MOESM1_ESM.pptx]

## Slide 1
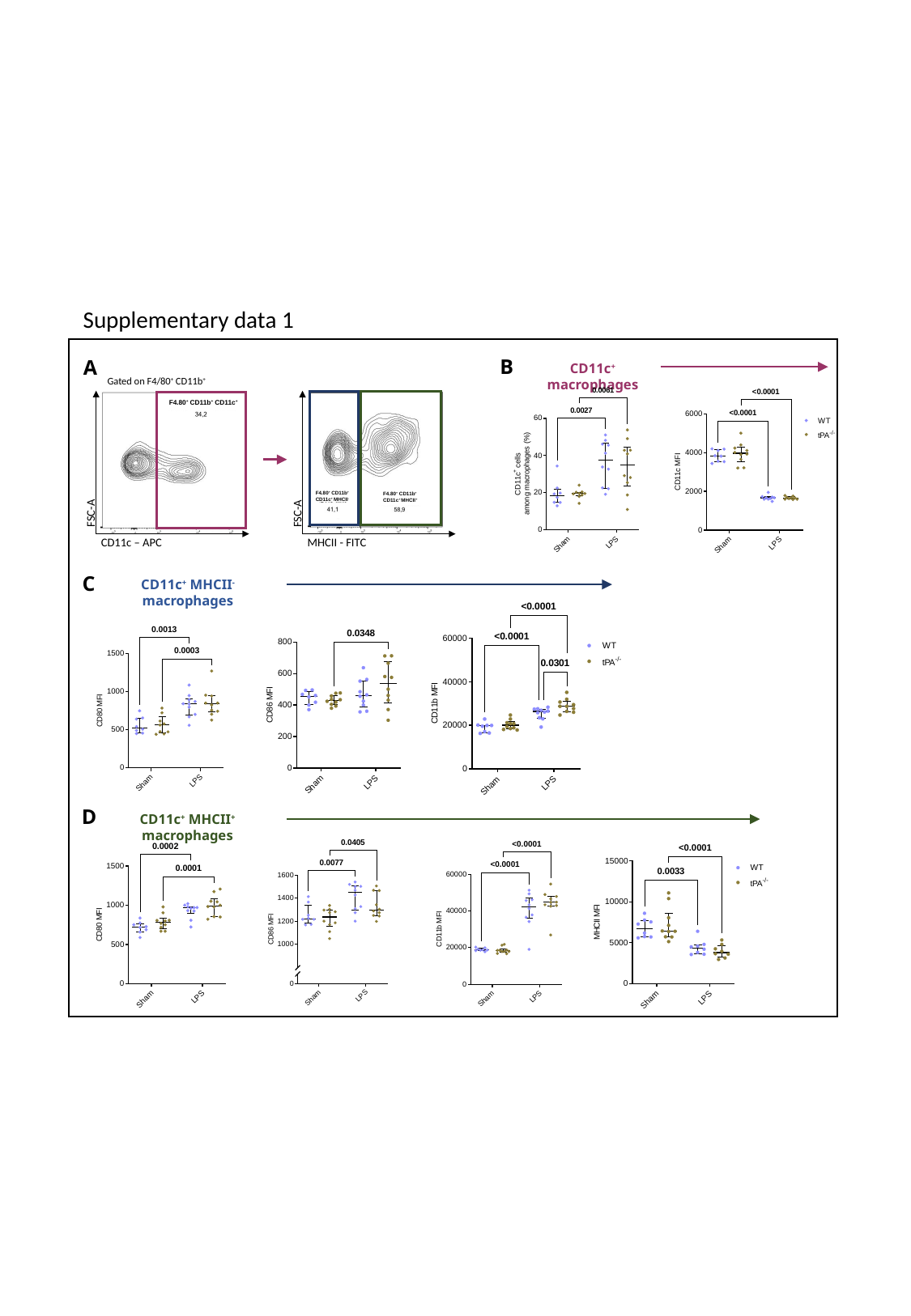

Supplementary data 1
B
A
CD11c+ macrophages
Gated on F4/80+ CD11b+
F4.80+ CD11b+ CD11c+
F4.80+ CD11b+ CD11c+ MHCII-
F4.80+ CD11b+ CD11c+ MHCII+
FSC-A
FSC-A
CD11c – APC
MHCII - FITC
C
CD11c+ MHCII- macrophages
D
CD11c+ MHCII+ macrophages

## Slide 2
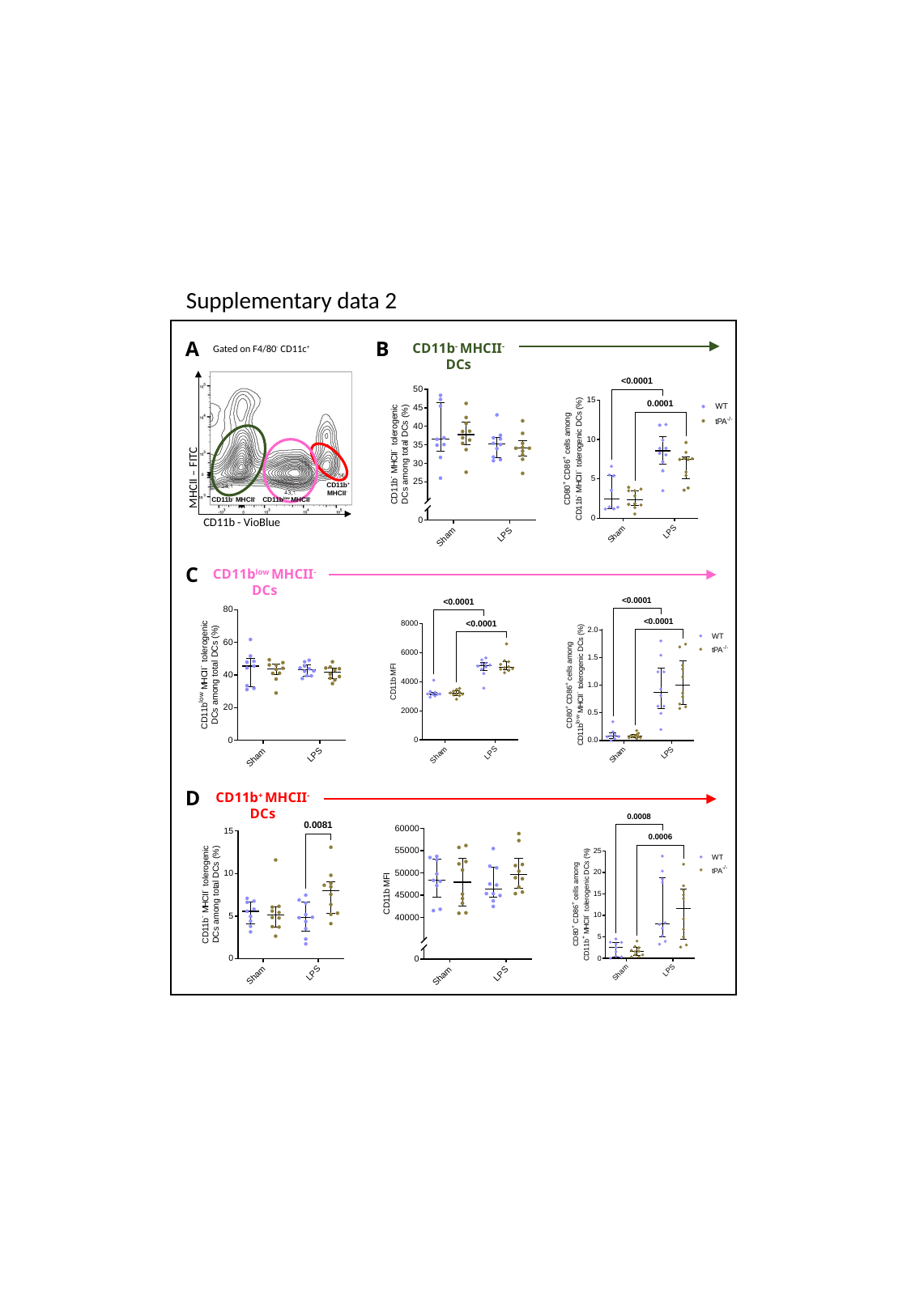

Supplementary data 2
A
B
CD11b- MHCII- DCs
Gated on F4/80- CD11c+
MHCII – FITC
CD11b - VioBlue
CD11b+
MHCII-
CD11blow MHCII-
CD11b- MHCII-
C
CD11blow MHCII- DCs
D
CD11b+ MHCII- DCs

## Slide 3
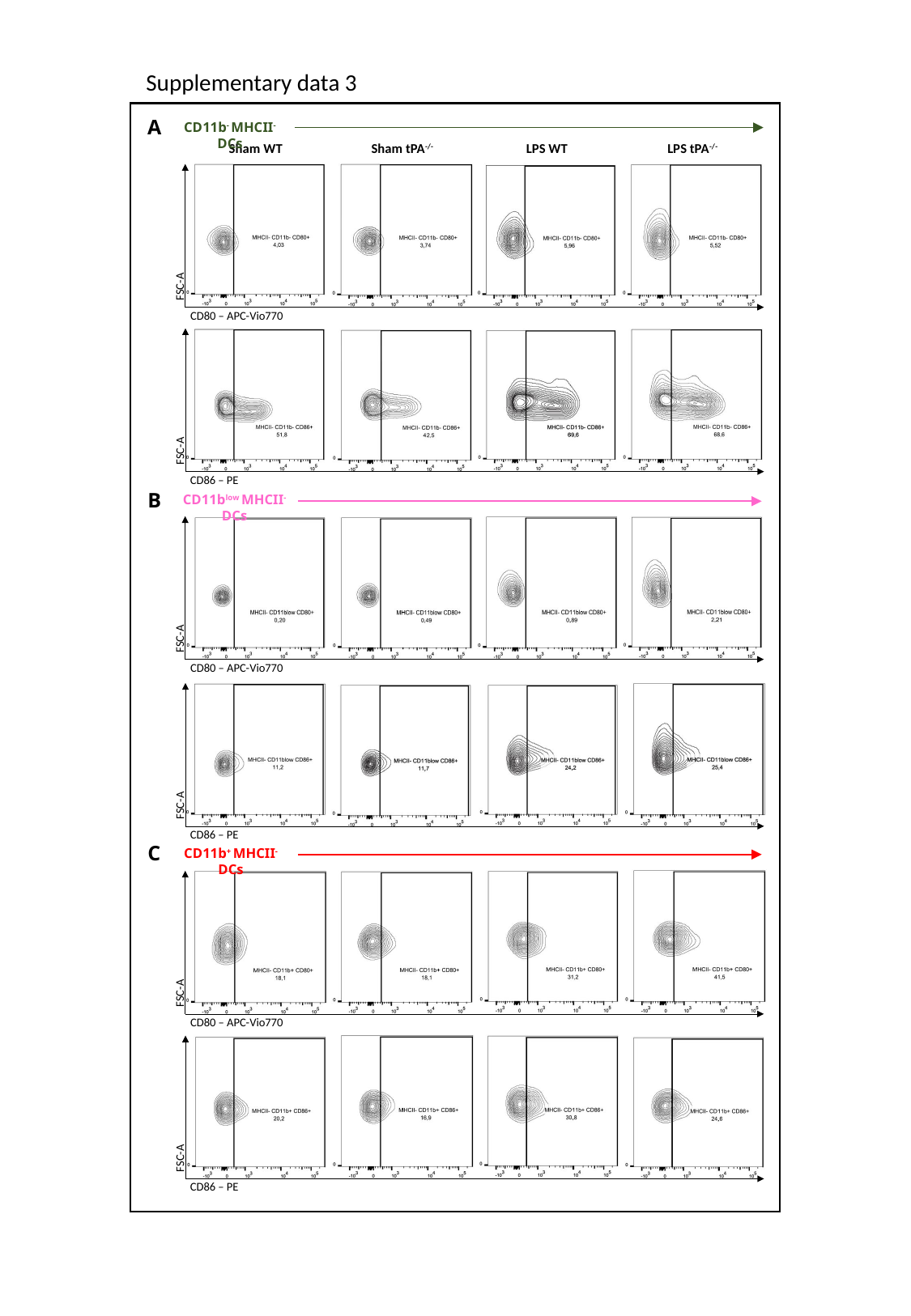

Supplementary data 3
A
CD11b- MHCII- DCs
LPS WT
LPS tPA-/-
Sham tPA-/-
Sham WT
FSC-A
CD80 – APC-Vio770
FSC-A
CD86 – PE
B
CD11blow MHCII- DCs
FSC-A
CD80 – APC-Vio770
FSC-A
CD86 – PE
C
CD11b+ MHCII- DCs
FSC-A
CD80 – APC-Vio770
FSC-A
CD86 – PE
